# Transvaginal uterine niche repair: surgical technique and outcome

**DOI:** 10.52054/FVVO.16.3.039

**Published:** 2024-09-30

**Authors:** D Coppenrath, D Timmerman, E De Jonge, H Van Kerrebroeck

**Affiliations:** Gynaecology and obstetrics, Ziekenhuis Oost Limburg, Genk, 3600, Belgium; Gynaecology and obstetrics, Catholic University, Leuven, 3000, Belgium; Gynaecology and obstetrics, Ziekenhuis Oost Limburg, Genk, 3600, Belgium

**Keywords:** Isthmocele, uterine niche, secondary subfertility, caesarean section, caesarean scar, vaginal repair

## Abstract

**Background:**

A uterine niche after caesarean section may play a role in secondary infertility. The transvaginal approach is a newly developed minimally invasive surgical technique for repairing a uterine isthmocele.

**Objectives:**

To report on the feasibility, effectiveness, and safety of the transvaginal uterine niche repair. The technique is demonstrated in a live-surgery video.

**Materials and Methods:**

A retrospective chart review involving all patients with secondary infertility who underwent a transvaginal uterine niche repair in Ziekenhuis Oost-Limburg between August 2019 and July 2022 was conducted.

**Main outcome measures:**

We compared the pre- and postoperative residual myometrial thickness as a primary surgical outcome measurement. The pregnancy ratio and the peri- and postoperative complications were also reported.

**Results:**

A total of 26 patients underwent a transvaginal uterine niche repair with an average operation time of 44 minutes. No major surgical complications were reported. 23 patients (88%) had good postoperative myometrial integrity, while 3 patients had a partial or complete postoperative recurrence of the uterine niche. The average pre- and postoperative myometrial thicknesses were 1.6 mm and 6.4 mm respectively. 64% of patients desiring pregnancy became pregnant after the transvaginal niche repair. There were no obstetric complications reported.

**Conclusions:**

A transvaginal approach is a safe and effective technique for uterine niche repair. It offers good results in re-establishing myometrial integrity and may favour fertility outcomes. It represents a valid minimal invasive procedure for patients with a very thin residual myometrial thickness and secondary infertility without leaving a visual scar.

## Introduction

After 50-70 percent of caesarean sections, a uterine iche appears. This happens when the uterine scar of the caesarean section heals insufficiently and an indentation of the myometrium develops (Budny-Winska and Pomorski, 2021). Risk factors for niche formation include multiple caesareans, caesarean during labour with low incision and a retroverted uterus (Torre et al., 2021). Due to the increasing number of caesarean sections, niche formation is becoming a more frequent problem.

A uterine niche can be diagnosed on ultrasound, magnetic resonance imaging, sonohysterography, or hysteroscopy (Budny-Winska and Pomorski, 2021). In the literature a uterine niche is often described as an indentation of ≥2mm of the myometrium at the site of the uterine incision (Torre et al., 2021).

A uterine niche is often asymptomatic, but potential symptoms include abnormal uterine bleeding, chronic pelvic pain, and secondary subfertility. These symptoms are often referred to as the ‘caesarean scar syndrome’ (Torre et al., 2021).

It is believed that due to the accumulation and adherence of blood and mucus in the isthmocele, inflammatory changes in the uterine cavity may adversely affect the implantation of an embryo by changing the endometrial receptivity. This blood and mucus may also cause implantation failure due to mechanical obstruction (Tanimura et al., 2015). Hsu et al. (2022) found a higher percentage of bacterial colonisation, especially Pseudomonas species, in patients with uterine niches and secondary infertility. In addition to secondary subfertility, a uterine niche can also cause rupture of the uterus by a subsequent pregnancy and can provoke difficulties during an embryo-transfer whereby the transfer is performed in the niche instead of the uterine cavity (Wang et al., 2020).

In general, a uterine niche is repaired by a laparoscopic or hysteroscopic approach. The reproductive outcome after hysteroscopic management of an isthmocele was assessed by Abdou and Ammar (2018) in a randomised trial. They showed a significantly higher pregnancy rate in the hysteroscopic-treated group versus the untreated group (75% versus 32 %, p = 0.001) (Abdou and Ammar, 2018). Although effective, the hysteroscopic technique does not excise the uterine scar and may further decrease the myometrial thickness. Furthermore, a residual myometrial thickness of at least 2 mm is necessary to ensure a safe procedure and pregnancy (Harjee et al., 2021; Mc Gowan et al., 2022).

Little data is available about the reproductive outcome after laparoscopic management. Although the systematic review of Harjee et al. (2021) showed a pregnancy rate of 50% after laparoscopic treatment of a uterine niche. These data suggest that the surgical repair of a uterine niche may be effective for the treatment of secondary subfertility.

In search of a less invasive method for uterine niche repair, the transvaginal approach was developed. This technique was reported for the first time by Candiani et al. (2019) and Mancuso et al. (2021) in a case report with video review. Both authors described the technique as an effective and safe method to repair uterine niche defects. It appears to be a minimally invasive technique with a quick postoperative recovery and without leaving a visual scar (Candiani et al., 2019).

## Materials and methods

For this retrospective cohort study, all patients who underwent a transvaginal uterine niche repair for unexplained secondary infertility in ZOL Genk between 08-08-2019 and 31-07-2022 were included for analysis. They all had a previous caesarean section and complained of unexplained secondary infertility or recurrent miscarriages for more than one year.

The uterine niche was diagnosed by transvaginal ultrasound by an experienced ultrasound examiner. Women with a maximal residual myometrial scar thickness of 3 millimetres were counselled for a transvaginal uterine niche repair.

**Figure 1:**
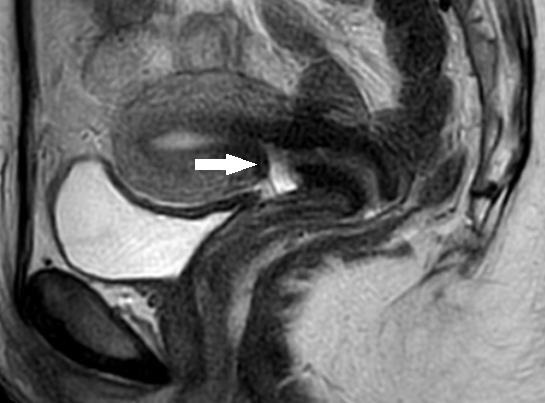
Preoperative MRI imaging. Legend: arrow indicating niche.

**Figure 2:**
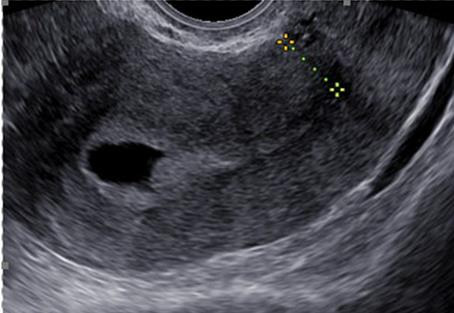
Postoperative ultrasound imaging.

The pre- and postoperative niche characteristics were analysed by the modified Delphi procedure (Jordans et al., 2019). Both the niche depth and the residual myometrium were measured. Most of the women who were eligible for a transvaginal niche repair had a pre-operative MRI (Figure 1). When available, MRI measurements were used for the data analysis. If not available, the measurements by ultrasound were used. A postoperative review of the niche was done by ultrasound three months after the surgery (Figure 2). The pre- and postoperative niche characteristics were compared by a paired t-test with a significance level of <0.05 using SPSS software (version 28.0.1.1, IBM).

This study was approved by the ethics committee of Ziekenhuis Oost-Limburg on 28 August 2022 with approval number Z-2022044. Given the retrospective data analysis, informed consent was not applicable.

### Transvaginal procedure: description of the technique

The surgery can be performed under spinal or general anaesthesia.

The patient is positioned in a dorsal lithotomy position and receives a single dose of prophylactic antibiotics cefazoline 2 grams intravenously.

Disinfection is achieved with povidone-iodine for gynaecological purposes and the patient is covered in sterile sheets.

For optimal visualisation, a Lone Star retractor is installed. A speculum is placed, and a Littlewood tissue forceps is attached on the cervix to provoke descensus. Figure 3: (A) Hydrodissection of the anterior peri-cervical endopelvic fascia with 10- 20cc diluted xylocaine/adrenaline 1% (B) A semi- circular incision in the anterior fornix is made. (C) The bladder is mobilised upwards by sharp dissection and the vesico-uterine peritoneal fold is opened.

**Figure 3A:**
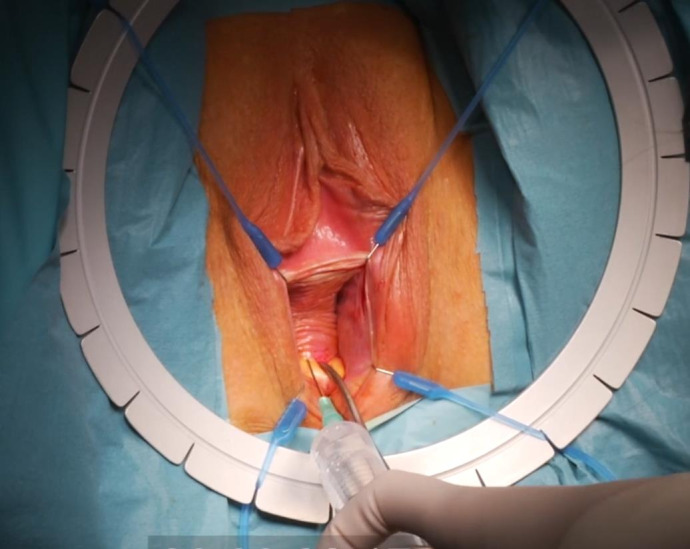
Transvaginal niche repair procedure: Hydrodissection of the anterior peri-cervical endopelvic fascia with 10-20cc diluted xylocaine/adrenaline 1%.

**Figure 3B:**
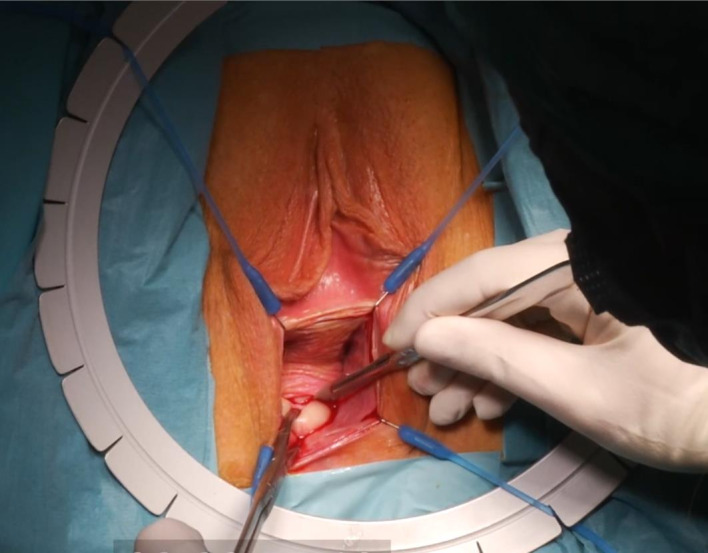
Transvaginal niche repair procedure: A semi-circular incision in the anterior fornix is made.

**Figure 3C:**
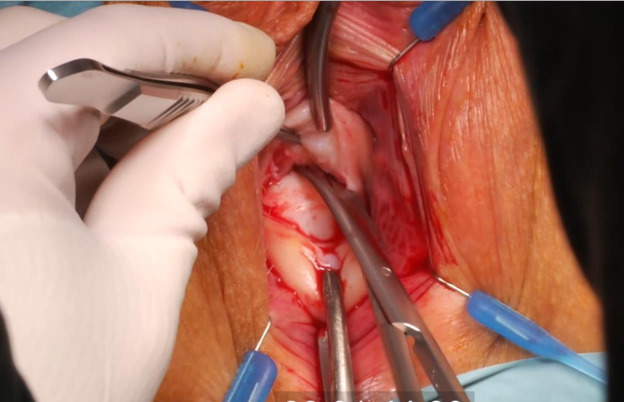
The bladder is mobilised upwards by sharp dissection and the vesico-uterine peritoneal fold is opened.

(D) A 3-mm hysteroscope is introduced in the cervix and by translucency the niche defect is marked. (E) The niche is resected with a scalpel to the edge of healthy full thickness myometrium. (F) One layer of separate vicryl 2/0 sutures is used to close the defect. When there is a very thick myometrium, a second layer of vicryl 2/0 stitches can be added. To protect the posterior uterine wall during suturing, a small lamella is placed in the uterine cavity. (G) The vaginal epithelium is closed with a continuous suture vicryl 2/0. (H) Immediate postprocedural hysteroscopic assessment confirms a patent cervical canal with easy access to the uterine cavity and a desirable repair of the uterine niche. Cystoscopy is performed to confirm an intact bladder mucosa.

**Figure 3D:**
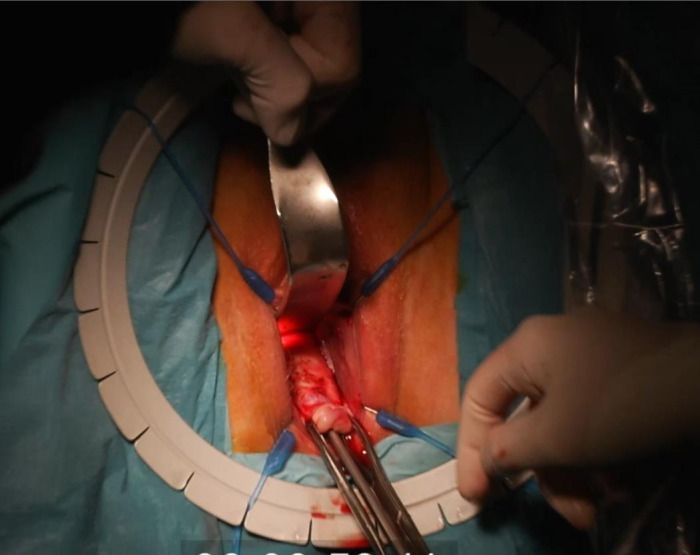
A 3-mm hysteroscope is introduced in the cervix and by translucency the niche defect is marked.

**Figure 3E:**
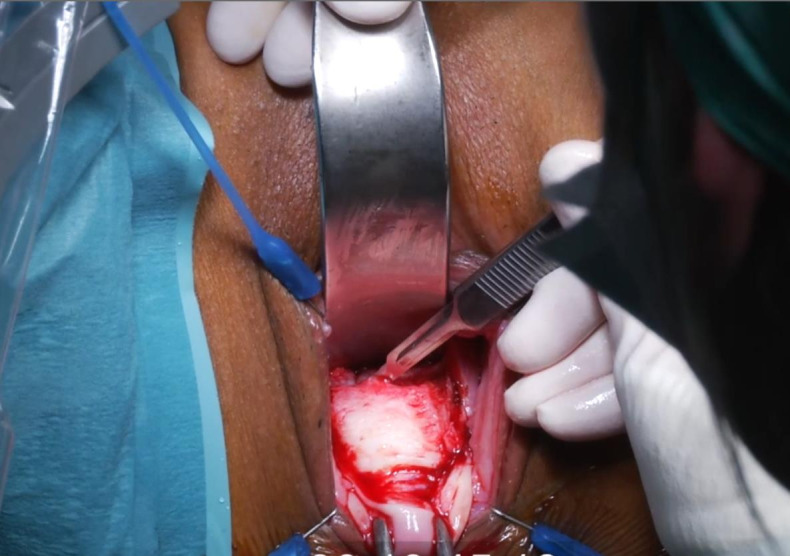
The niche is resected with a scalpel to the edge of healthy full thickness myometrium.

**Figure 3F:**
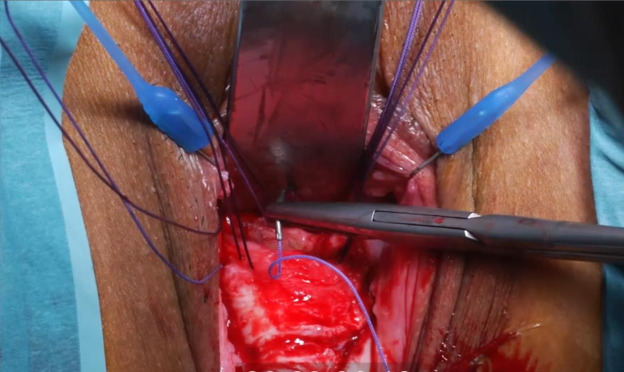
One layer of separate vicryl 2/0 sutures is used to close the defect. In case of a very thick myometrium, a second layer of vicryl 2/0 stitches can be added. To protect the posterior uterine wall during suturing, a small lamella is placed in the uterine cavity.

**Figure 3G:**
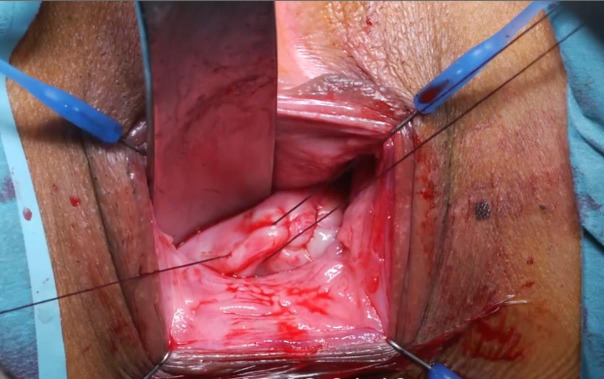
The vaginal epithelium is closed with a continuous suture vicryl 2/0.

**Figure 3H:**
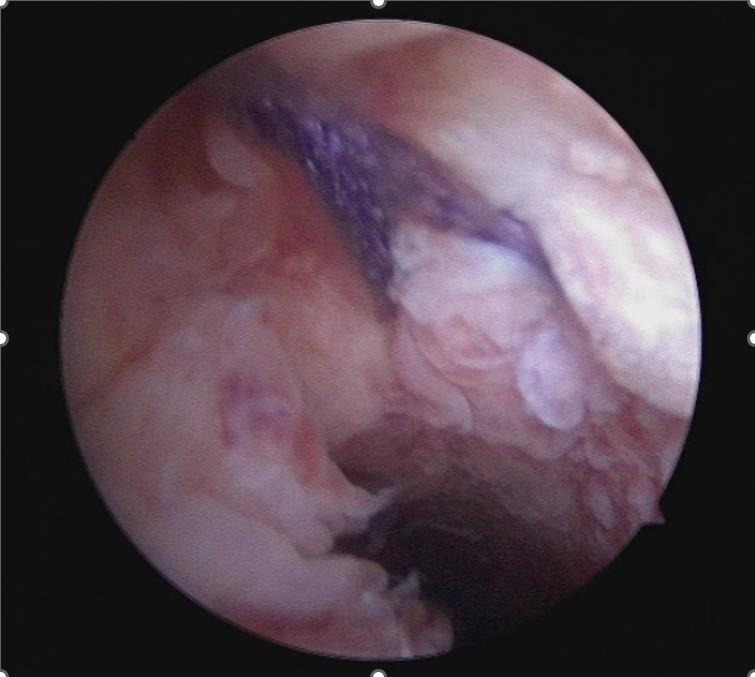
Immediate postprocedural hysteroscopic assessment confirms a patent cervical canal with easy access to the uterine cavity and a desirable repair of the uterine niche.

For a full review of the technique, a video of the surgical procedure is available on: https://qrco.de/bfOBLB

## Results

In the 35-month study period, we performed 26 transvaginal niche repairs for unexplained secondary infertility. All patients had multiple unsuccessful in vitro fertilisation treatments or more than one miscarriage prior to the uterine niche repair. Table I refers to the patient characteristics. A significant improvement in the myometrial integrity of the lower uterine segment was obtained using this technique. The pre-and postoperative measurements of the niche depth and residual myometrial thickness are presented in Table II. Three months postoperative 23 patients (88%) had good myometrial integrity with a thickness of more than 3mm. Nevertheless, three patients had a partial or complete postoperative recurrence of the uterine niche. One of these patients had a uterine defect high up in the lower segment which in fact was difficult to reach with the transvaginal approach. Another patient with a substandard result had an associated myoma in the vicinity of the uterine scar which was probably detrimental to good postoperative healing. The third case had no recognised potential risk factor for inadvertent wound healing. As shown in Table III, the procedure is safe and effective. One patient developed a partial stenosis of the cervix that was restored by hysteroscopic resection at three months postoperatively. However, the healing of the uterine scar in this case was adequate and no residual niche was observed. No patients complained about dyspareunia.

**Table I:** Patient characteristics.

Age	35.3 (30-45)
BMI	24.1 (19-34)
Time between c-section and repair (months)	45.8 (13-120)
One C-section	81%
Two C-sections	19%

**Table II:** Niche characteristics.

Preoperative	Postoperative	p-value
Niche depth (mm)	7.2 (4-10)	2.2 (0-9)	<0.001
Myometrial thickness (mm)	1.6 (0.5-3.0)	6.4 (1-12)	<0.001

**Table III:** Short- and long-term outcome of surgery.

Average duration of hospital stay (days)	0.95 (0.5-1)
Average estimated blood loss (ml)	57 (50-200)
Average operation time (min)	48 (30-60)
Healthy pregnancy ratio	64%
Average time until healthy pregnancy (months)	12.6
Percentage spontaneous pregnancy	56%
Percentage IVF pregnancy	44%
Obstetric complications	0

We advised our patients to wait at least six months after the surgery before getting pregnant. After this time, one patient had no pregnancy wishes anymore. Of the other 25 women who attempted to become pregnant after the surgery, 16 (64%) achieved this. Nine women became pregnant spontaneously and seven women by in vitro fertilisation. At this point in time twelve patients delivered a healthy baby and in four patients the pregnancy is ongoing. There were no obstetric complications reported. All patients underwent a primary caesarean section at a gestational age between 38 and 39 weeks.

## Discussion

This study reports on the first 26 transvaginal niche repairs that were performed in our fertility unit. As we gained more experience, the ease of the surgery grew, and the duration of the procedure decreased. The average operation time in the last 13 vaginal niche repairs was six minutes less in comparison with the first 13 procedures. This is indicative of a procedure with a short learning curve and was shown to be safe and effective in restoring myometrial integrity (Candiani et al., 2019; Mancuso et al., 2021). In our analysis, there was a significant decrease in niche depth and a significant increase in myometrial thickness three months postoperatively. No patients complained about dyspareunia postoperatively.

Vitale et al. (2020) compared hysteroscopic, laparoscopic, and vaginal repair in a systematic review. All techniques seem to be a good option for treating symptomatic uterine isthmocele. Hysteroscopic correction is the safest option in patients with adequate residual myometrial thickness. Although laparoscopic or vaginal repair is preferred when the residual myometrial thickness is less than 2.5 to 3 mm. A hysteroscopic repair is not preferred in this case due to the higher risk of uterine perforation and bladder injury. Besides, by complete excision of the scar tissue in a laparoscopic or vaginal repair, the uterine wall is strengthened, which can be important in an upcoming pregnancy. To determine the preferred surgical intervention for a uterine niche, adequate evaluation of the overlying myometrial thickness is required (Vitale et al., 2020).

There is currently no evidence supporting the superiority of a laparoscopic repair over a transvaginal repair (Vitale et al., 2020). However, the transvaginal approach is easier when the defect is located low in the uterine segment. A high defect is more difficult to reach and may possibly be more suitable for a laparoscopic repair. The decision of the surgical intervention should depend on the expertise of the surgical team. A randomised controlled trial to compare a transvaginal niche repair and a laparoscopic repair is necessary to evaluate the possible advantages and disadvantages of these surgical techniques.

Transvaginal ultrasound in combination with MRI imaging was routinely used to explore the location, extension, and properties of the uterine niche. Both ultrasound and MRI were accurate and corresponded in the evaluation of the myometrial integrity. Currently, there are no guidelines that determine the need to perform MRI imaging to investigate a uterine niche. As stated by Armstrong et al. (2023) MRI is slightly more accurate in the measurement of the residual myometrial thickness, and it can be useful for research purposes because of the decreased interobserver variability.

A recent meta-analysis by Vitagliano et al. (2024) described the negative impact of an isthmocele on the success rate of in vitro fertilisation. When intracavitary fluid is present, the uterine niche is associated with poorer IVF outcomes (Vitagliano et al., 2024).

Although little evidence is available regarding the effect of a niche repair on secondary infertility, there are small observational studies assessing transvaginal niche repair and pregnancy. A small study by Zhou et al (2018) showed a healthy pregnancy rate of 35.3% with a follow-up of 15 months after the vaginal niche repair. In a larger retrospective study by Deng et al. (2021) the healthy pregnancy rate after a vaginal niche repair was 58%. In our study, the healthy pregnancy rate was 64%. Despite these promising results, we only analysed 26 cases of transvaginal niche repairs. A larger study population is necessary to further explore the risks and benefits of these procedures. Besides these promising results, no randomised controlled trials are available to determine the true effect of a niche repair compared to waiting for a spontaneous pregnancy without surgical treatment. A possible bias in these studies is that patients often undergo fertility procedures like hysterosalpingo-foam sonography (HyFoSy) which can also improve fertility outcomes (De Neubourg et al., 2021).

## Conclusion

The transvaginal approach is a safe and effective technique for uterine niche repair. In comparison with a laparoscopic approach, it is a less invasive procedure without leaving a visual scar. It is also an option for patients with a very thin residual myometrial thickness where a safe hysteroscopic repair is not possible. A vaginal niche repair offers good results in re-establishing myometrial integrity and may favour fertility outcomes. Larger studies are needed to confirm its safety and effectiveness and to determine the potential advantages and disadvantages of a transvaginal repair compared to a laparoscopic approach.
